# A commentary on “Comparison of surgical outcomes and learning curve for robotic versus laparoscopic living donor hepatectomy: a retrospective cohort study” (*Int J Surg* 2022; 108: 107000)

**DOI:** 10.1097/JS9.0000000000000294

**Published:** 2023-03-24

**Authors:** Shi-Yun Zhong, Zhi-Peng Liu, Yi-Shi Yang, Ting Yu, Xiao-Jun Wang

**Affiliations:** Department of Hepatobiliary Surgery, Southwest Hospital, Third Military Medical University (Army Medical University), Chongqing, China

*Dear Editor*,

The article by Dr Kim *et al*.^1^ was very interesting. This article provided the first analysis in which two minimally invasive surgical techniques, laparoscopic living donor right hemihepatectomy (LLDRH) and robotic living donor right hemihepatectomy (RLDRH), were compared for living donor hepatectomy at a single center. The author found that RLDRH resulted in less intraoperative bleeding but had comparable postoperative outcomes to LLDRH. Moreover, donors with hilar structure anatomic variation were more likely to undergo RLDRH. In addition, the standardized procedures for RLDRH might help set up pure minimally invasive procedures and facilitate the safe implementation of laparoscopic approaches. Despite being inspiring and thought-provoking, we have the following comments:

First, the authors used moving average charts for graft-out time and a cumulative sum (CUSUM) analysis to assess the learning curves for RLDRH and LLDRH. The results showed that LLRDH stabilized earlier than RLDRH. The authors recommended LLRDH be performed based on the experience of RLDRH and the same surgical technique because of the difference in duration between the two procedures. However, we believe that the learning curves may not be accurate enough. To be frank, the most important process for RLDRH and LLDRH is partial hepatectomy. In addition to being exposed to this operation during transplantation, surgeons might also use liver resection skills in other operations, such as tumor resection, which is also a learning process for the operator. Therefore, we believe that only including the data of minimally invasive donor hepatectomy as the establishment of learning curve may cause the learning curve to be less accurate.

Second, to reduce the bias in patient selection, the authors compared the outcomes of RLDRH and LLDRH in subgroups after 2019. We know that the surgeon in the RLDRH group had already completed 3 years of training before 2019, while that in the LLDRH group had just begun. Patients undergoing living hepatectomy are generally healthy people; thus, we speculate that the surgical approach and the surgeon’s skills are more likely to influence the short-term outcome.

Third, the learning curves for both RLDRH and LLDRH showed a tendency to be unstable initially. But they gradually stabilized as the number of procedures performed increased. We hypothesized that if the authors had performed a subgroup comparison of patients after the learning curve had stabilized, they might reduce the bias due to differences in surgical proficiency.

Finally, we are not questioning the relevancy of this impactful team study by presenting these comments reflecting some personal opinions we want to communicate with the authors. We appreciate the authors and the *International Journal of Surgery* for publishing this clinically significant study that will advance the development and progress of living donor hepatectomy.

## Ethical approval

Not available.

## Sources of funding

Chongqing Technology Innovation and Application Development Special Key Project (No. CSTC2021jscx-gksb-N0009).

## Author contribution

S.-Y.Z., Z.-P.L., Y.-S. Y., T. Y.: manuscript preparation. X.-J. W.: critical revision. Final approval of manuscript is done by all authors.

## Conflict of interest disclosure

All authors have declared no conflict of interest.

## Guarantor

Xiao-Jun Wang.
